# Peripheral blood neurotrophic factor levels in children with autism spectrum disorder: a meta-analysis

**DOI:** 10.1038/s41598-020-79080-w

**Published:** 2021-01-08

**Authors:** Shu-Han Liu, Xiao-Jie Shi, Fang-Cheng Fan, Yong Cheng

**Affiliations:** Center On Translational Neuroscience, College of Life and Environmental Sciences, Minzu University of China, 27 South Zhongguancun Avenue, Zhongguancun South St, Haidian District, Beijing, 100081 China

**Keywords:** Neurotrophic factors, Autism spectrum disorders, Diseases

## Abstract

Increasing evidence suggests that abnormal regulation of neurotrophic factors is involved in the etiology and pathogenesis of Autism Spectrum Disorder (ASD). However, clinical data on neurotrophic factor levels in children with ASD were inconsistent. Therefore, we performed a systematic review of peripheral blood neurotrophic factors levels in children with ASD, and quantitatively summarized the clinical data of peripheral blood neurotrophic factors in ASD children and healthy controls. A systematic search of PubMed and Web of Science identified 31 studies with 2627 ASD children and 4418 healthy controls to be included in the meta-analysis. The results of random effect meta-analysis showed that the peripheral blood levels of brain-derived neurotrophic factor (Hedges’ g = 0.302; 95% CI = 0.014 to 0.591; *P* = 0.040) , nerve growth factor (Hedges’ g = 0.395; 95% CI = 0.104 to 0.686; *P* = 0.008) and vascular endothelial growth factor (VEGF) (Hedges’ g = 0.097; 95% CI = 0.018 to 0.175; *P* = 0.016) in children with ASD were significantly higher than that of healthy controls, whereas blood neurotrophin-3 (Hedges’ g =  − 0.795; 95% CI =  − 1.723 to 0.134; *P* = 0.093) and neurotrophin-4 (Hedges’ g = 0.182; 95% CI =  − 0.285 to 0.650; *P* = 0.445) levels did not show significant differences between cases and controls. Taken together, these results clarified circulating neurotrophic factor profile in children with ASD, strengthening clinical evidence of neurotrophic factor aberrations in children with ASD.

## Introduction

Autism Spectrum Disorder (ASD) refer to a group of neurodevelopmental disorders characterized primarily by restrictive, repetitive patterns of behaviors, loof of interest or activity, and social communication impairment. The disease includes autistic disorder, Asperger's syndrome and pervasive developmental disorder-not otherwise specified^1,2^. According to the Autism and Developmental Disabilities Monitoring Network, the number of children diagnosed with ASD has increased by 150% since 2000, with the overall prevalence of ASD estimated at 16.8 per 1000 children aged 8 years in 2014^3^. Twin studies have shown that the concordance of ASD in monozygotic twins is higher than that in dizygotic twins, suggesting that ASD is highly heritable^4^. However, the coincidence rate between identical twins and autism and related diseases was less than 100%, indicating that environmental factors also play a role in ASD^5^.

The neurotrophins are a family of proteins that have been shown to play an important role in the central and peripheral nervous system, which control a number of aspects of survival, development, and function of neurons^6^. The so-called “classic” neurotrophin family includes nerve growth factor (NGF), brain-derived neurotrophic factor (BDNF), neurotrophin-3 (NT-3) and neurotrophin-4 (NT-4)^7^. Recently, research findings from analyses of postmortem and peripheral tissue and molecular genetic studies led to the hypothesis that neurotrophins—as crucial regulators of neuroplasticity—impacting on the pathophysiologic features of ASD^8^. In a trios-based association study, Nishimura et al. showed that the BDNF SNP haplotype combinations significantly associated with ASD^9^. Moreover, Postmortem studies found elevated BDNF protein levels in the fusiform gyrus tissue^10^ and elevated NT-3 protein levels in the cerebellum^11^ from patients with ASD. However, molecular genetic studies and postmortem studies on neurotrophic factors were scarce, and therefore it is difficult to evaluate the robustness of the associations of neurotrophins with ASD.

Increasing number of clinical studies measured peripheral blood levels of neurotrophic factors in children with ASD, due to the easy accessibility of blood and “periphery as a window to the brain” hypothesis^12^. However, clinical data on neurotrophic factor levels in children with ASD have yielded inconsistent results. One study showed that the serum BDNF levels of children with ASD were significantly higher than that of the control subjects^13^, whereas Makkonen et al.^14^ suggested that was no significant difference in serum BDNF concentrations between cases and controls, and another study showed that BDNF serum levels were significantly decreased in ASD children when compared with controls^15^. For VEGF, results from Emanuele et al. showed that VEGF levels in patients with ASD were lower than that of healthy controls^16^. In contrast, one study indicated that VEGF levels did not show significantly difference between children with ASD and healthy controls^17^. Moreover, studies have demonstrated inconsistent data for IGF-1 and IGF-2 levels comparing ASD children and healthy controls^18,19^. Given the inconsistent findings on neurotrophic factors in children with ASD, a meta-analysis on this subject is necessary.

To clarify neurotrophic factor profile in children with ASD, here we undertook a meta-analysis of studies measuring neurotrophic factor levels in blood of children with ASD and healthy controls.

## Results

**Figure 1:**
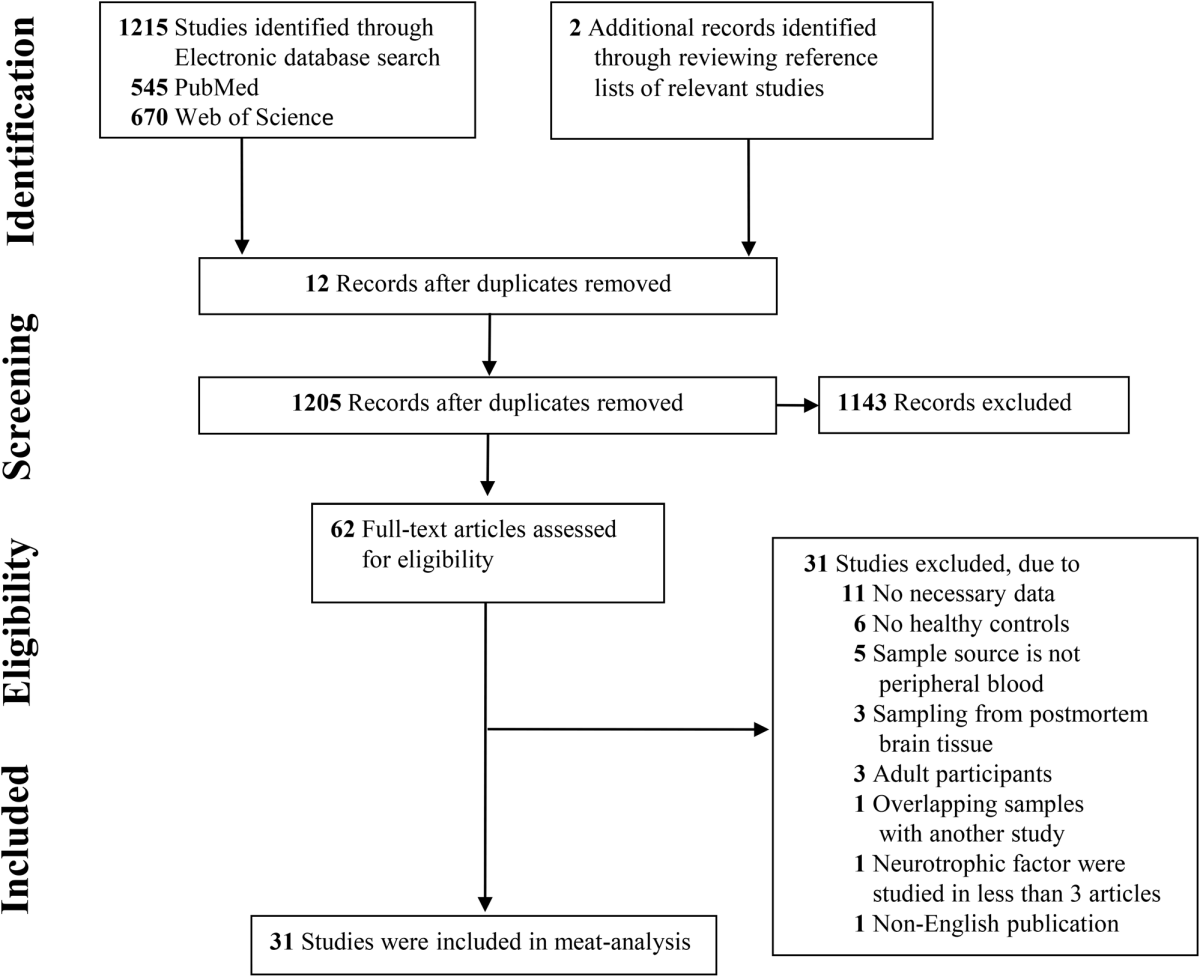
PRISMA flowchart of the literature search.

The initial search generated a total of 1217 records: 545 were searched from PubMed database, 670 from Web of Science and 2 additional records identified from the reference lists of relevant studies. After scanning the titles and abstracts, 62 articles relevant to present subject were identified for full-text scrutiny. Several studies were excluded as they did not have necessary data (11 studies)^20–30^; lack of healthy controls (6 studies)^31–36^; sample source is not peripheral blood (5 studies)^18,37–40^; samples derived from postmortem brain (3 studies)^10,11,41^; participants were adults (3 study)^16,42,43^; had patient samples that overlapped with another studies (1 study)^44^; neurotrophic factors were studied in less than 3 articles (1 study)^19^ and non-English publication (1 studies)^45^. Therefore, a total of 31 studies met the criteria were included for this meta-analysis (Fig. 1)^8,12,13,15,17,46–71^. Demographic and clinical profile of the included studies were presented in Supplementary Table S1.

### Main association of peripheral blood neurotrophic factor levels with ASD in children

**Figure 2:**
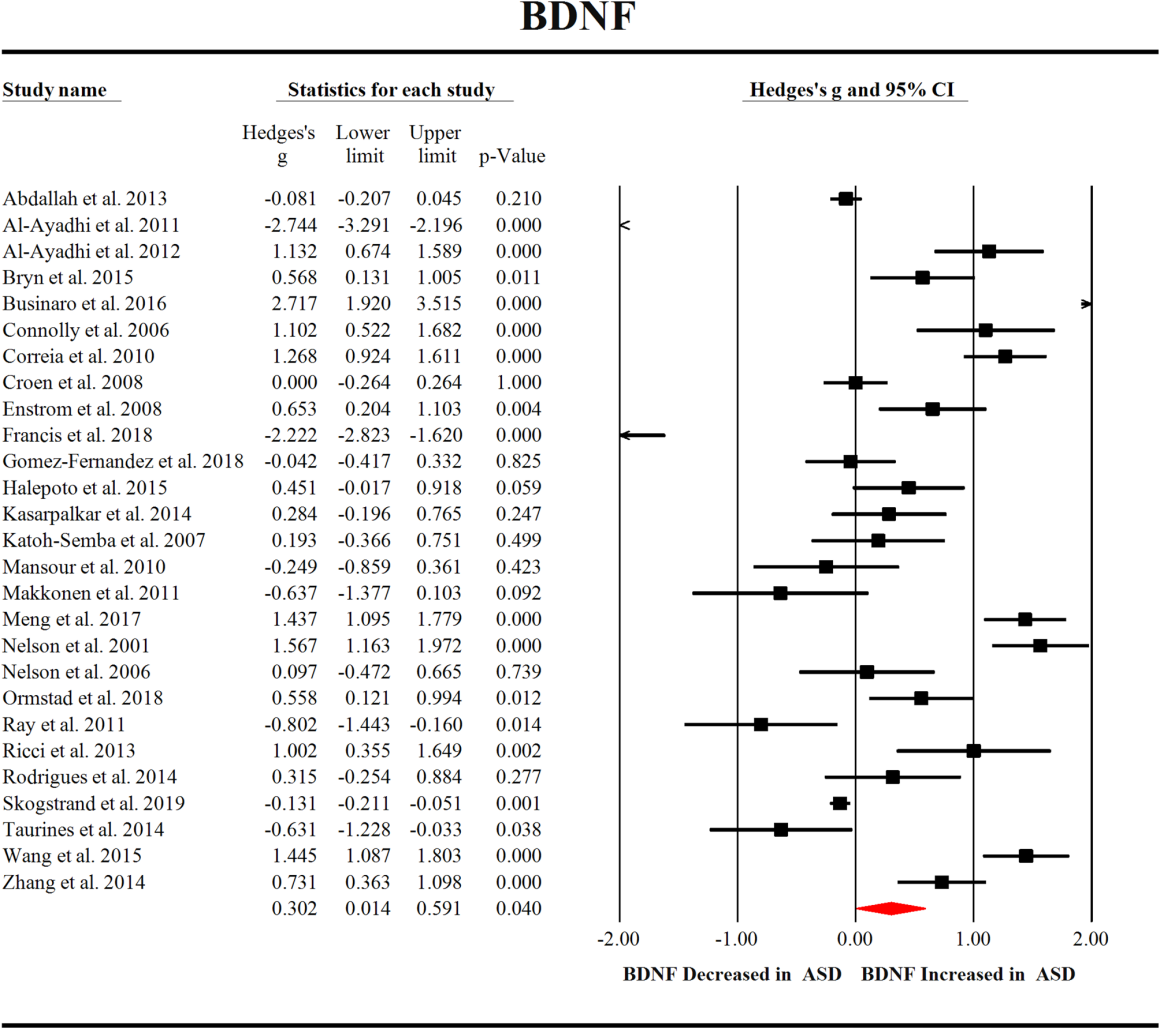
Forest plot for random-effects meta-analysis for BDNF. Forest plot for random-effects meta-analysis on differences in blood BDNF concentrations between children with autism spectrum disorder (ASD) and healthy controls. The sizes of the squares are proportional to study weight. Diamond marker indicates pooled effect size. CI, confidence interval.

**Figure 3:**
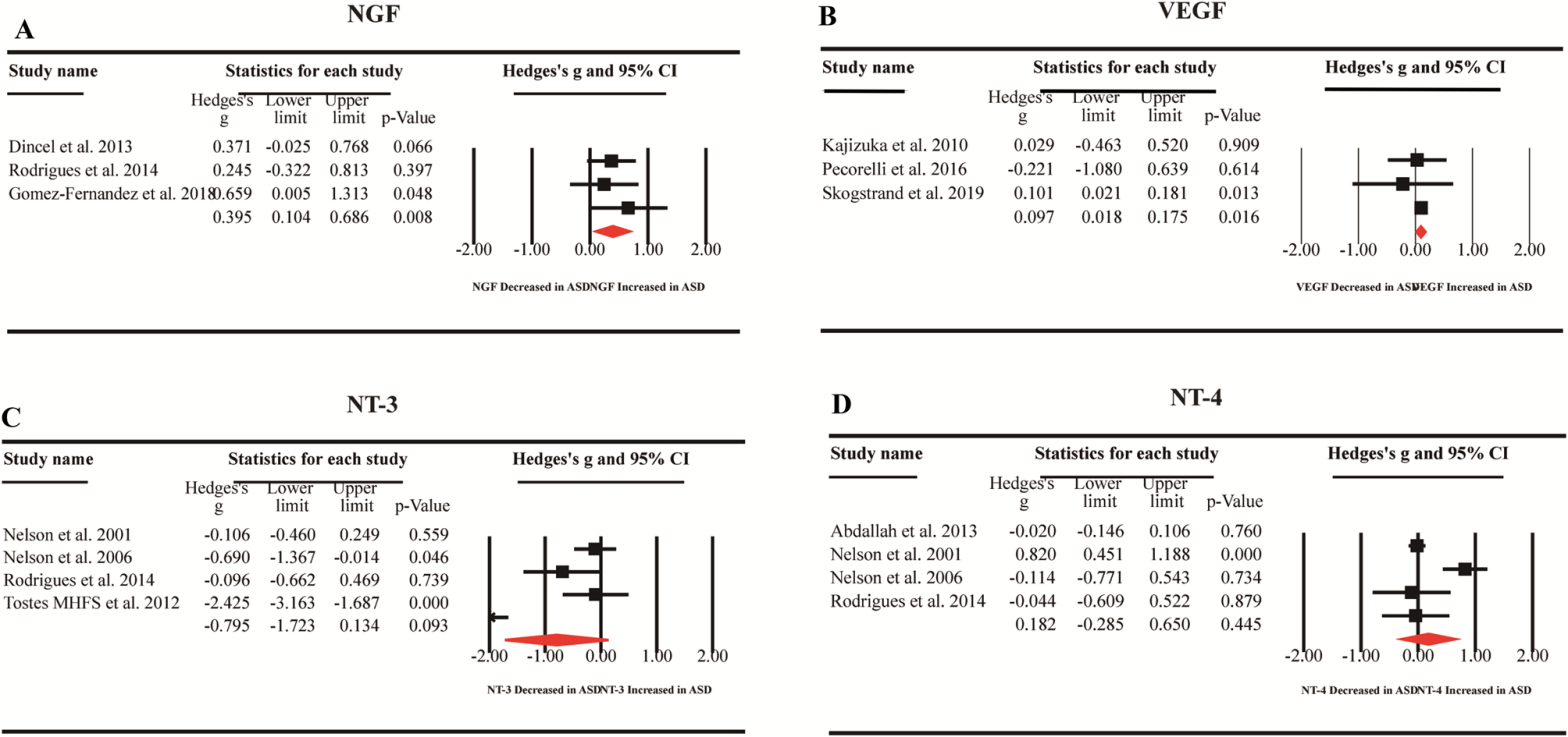
Forest plot for random-effects meta-analysis for NGF, VEGF, NT-3 and NT-4. Forest plot for random-effects meta-analysis on differences in blood NGF (**A**), VEGF (**B**), NT-3 (**C**) and NT-4 (**D**) concentrations between children with autism spectrum disorder (ASD) and healthy controls. The sizes of the squares are proportional to study weight. Diamond marker indicates pooled effect size. CI, confidence interval.

We first compared the peripheral blood BDNF levels between 2380 children with ASD and 4191 healthy controls extracted from 27 studies. Random-effects meta-analysis showed that ASD children had significantly increased levels of BDNF compared with healthy controls (Fig. 2, Hedges’ g = 0.302; 95% CI = 0.014 to 0.591; *P* = 0.040). Then, we compared blood NGF levels between 100 children with ASD and 84 healthy controls extracted from 3 studies, and the results showed that compared with healthy controls, children with ASD had significantly increased NGF levels (Fig. 3A, Hedges’ g = 0.395; 95% CI = 0.104 to 0.686; *P* = 0.008). In addition, we compared the blood VEGF levels of 844 children with ASD and 2460 healthy controls extracted from 3 studies, which showed significantly increased VEGF levels in ASD children when compared with healthy controls (Fig. 3B, Hedges’ g = 0.097; 95% CI = 0.018 to 0.175; *P* = 0.016). However, we did not observe significant differences between children with ASD and healthy controls for peripheral blood NT-3 (Hedges’ g =  − 0.795; 95% CI =  − 1.723 to 0.134; *P* = 0.093) and NT-4 (Hedges’ g = 0.182; 95% CI =  − 0.285 to 0.650; *P* = 0.445) levels (Fig. 3C,D).

### Investigation of heterogeneity

Of the three neurotrophic factors that were significantly associated with ASD, NGF and VEGF did show no significant between-study heterogeneity, whereas BDNF showed high levels of heterogeneity. Therefore, we next performed subgroup analyses to investigate potential source of the high levels of heterogeneity for studies measuring BDNF concentrations.

The sources of heterogeneity include category variables (sample source, assay type and medication status) and continuous variables (sample size, age, sex, publication year, course of disease and disease severity). Due to a substantial lack of information regarding medication status, course of disease and disease severity, we only performed subgroup analyses on sample sources (serum and plasma) and assay types (ELISA and no ELISA), as well as meta-regression analyses based on sample size, age, sex and publication year.

Subgroup analyses suggested that sampling source did not address the between-study heterogeneity, and we still observed significant heterogeneity for studies plasma (Q = 43.966; I^2^ = 88.628; *P* < 0.001) and serum (Q = 344.797; I^2^ = 95.650; *P* < 0.001) BDNF levels (see Supplementary Fig. S1). Additionally, subgroup analyses stratified by assay type showed increased BDNF levels in ASD children when compared with controls in ELISA group (20 studies, Hedges’ g = 0.404; 95% CI = 0.024 to 0.785; *P* = 0.037), but not in non-ELISA group (7 studies, Hedges’ g = 0.015; 95% CI =  − 0.618 to 0.647; *P* = 0.964). Again, we observed high levels of heterogeneity among studies for ELISA group (Q = 413.794; I^2^ = 95.408; *P* < 0.001) and non-ELISA group (Q = 119.886; I^2^ = 94.995; *P* < 0.001) (see Supplementary Fig. S2).

Meta-regression analyses showed that sample size, age, gender and publication year had no moderating effects on the outcome of the meta-analysis (*P* > 0.05 in all analyses) (see Supplementary Fig. S3).

Visual inspection of funnel plots suggested no risk of publication bias for studies analyzing BDNF, NGF and VEGF levels, and these were confirmed by the results of the Egger’s test (see Supplementary Fig. S4 and Table 1).

**Table 1:** Summary of comparative outcomes for measurements of neurotrophic factor levels.

TF	No. of studies	No. with ASD/controls	Main effect			Heterogeneity			Publication bias		
			Hedges g (95% CI)	Z score	*P* value	Q statistic	df	*P* value	I^2^ Statistic	Egger intercept	*P* value
BDNF	27	2380/4191	0.302 (0.014 to 0.591)	2.054	0.040	537.149	26	0.000	95.160	2.13562	0.11851
NGF	3	100/84	0.395 (0.104 to 0.686)	2.662	0.008	0.909	2	0.635	0.000	1.13092	0.71027
NT-3	4	140/114	− 0.795 (− 1.723 to 0.134)	− 1.678	0.093	33.086	3	0.000	90.933	− 7.11871	0.26344
NT-4	4	485/838	0.182 (− 0.285 to 0.650)	0.764	0.445	18.275	3	0.000	83.585	1.37770	0.61376
VEGF	3	844/2460	0.097 (0.018 to 0.175)	2.414	0.016	0.611	2	0.737	0.000	− 0.59890	0.23760

df, degrees of freedom; ASD, Autism Spectrum Disorder; BDNF, Brain-Derived Neurotrophic Factor; NGF, Nerve Growth Factor; NT-3, Neurotrophin-3; NT-4, Neurotrophin-4; VEGF, Vascular Endothelial Growth Factor; NTF, Neurotrophic Factor.

## Discussion

In this study, we performed a comprehensive investigation on the changes of peripheral neurotrophic factors in children with ASD. We included 31 studies with 2486 ASD children and 4303 healthy controls measuring five neurotrophic factors, and reported the levels of BDNF, NGF and VEGF were elevated in children with ASD. However, no significant associations were found between NT-3 or NT-4 and ASD. In addition, we found high levels of between-study heterogeneity for BDNF, whereas NGF and VEGF did not show between-study heterogeneities. We further performed subgroup analysis based on the sample source and assay type, and meta-regression analysis based on sample size, age, gender and publication year for studies measuring BDNF levels. However, we did not find potential sources of heterogeneity. This is consistent with a previous meta-analysis on blood BDNF levels in ASD children, which included a relatively small number of studies published in 2016^72^. Although studies from the literature in the past decade has produced inconsistent results, and the role of neurotrophic factors in children with ASD is still unclear, this study provides strong clinical evidence that the levels of BDNF, NGF and VEGF in peripheral blood of children with ASD are higher than those in healthy controls, strengthening the clinical evidence that neurotrophic factor plays a critical role in ASD onset and/or development.

Despite the largely unknown etiology and pathogenesis of ASD, researches have consistently demonstrated that children with ASD are accompanied by abnormal brain development^73,74^. Courchesne et al. investigated the neural basis of brain overgrowth in ASD children at the cellular level and found abnormal increases in the number of neurons in the prefrontal cortex of ASD boys^75^. Additionally, it has been suggested that that the brain volume overgrowth in early ASD children is mainly due to the increased proliferation of neural progenitor cells^76^. Neurotrophic factors play a positive role on the proliferation of embryonic neural progenitor cells^27^, and an in vivo study showed that BDNF increased neurogenesis in the granule cell layer of hippocampus in rats^77^. Importantly, BDNF levels are temporally regulated during development, which has been suggested to be required for proper neuronal development and functions^78^. Therefore, it is very likely that in the early stage of ASD children, abnormal regulation of BDNF leads to the subsequent long-term changes in the brain structure and function. Furthermore, it has been reported the excessive brain growth in ASD children occur prior to most clinical manifestations of the disease^79^, raising the possibility that the observed BDNF aberrations in ASD children found in the meta-analysis contributed to the excessive growth in brains of early ASD children.

We noted that levels of two other neurotrophic factors, NGF and VEGF, also increased compared with healthy controls. VEGF is a key signaling molecule of the central nervous system which is involved in neuroprotection, neuronal survival and axonal growth^80^. Similarly, NGF is also involved in important aspects of nerve cell growth, differentiation, survival, and regeneration, and thought may represent a serological marker for autistic children^23,81^. It is possible that that the structural changes in the brains of children with ASD may also be contributed by the abnormalities of NGF and VEGF. Therefore, targeting the neurotrophic factor system may provide a novel strategy for the potential treatment of ASD, and future studies are needed to validate the hypothesis. However, since the studies included in this meta-analysis analyzed NGF levels by ELISA method, and the commercially available ELISA kits could not differentiate between pro and mature forms of NGF, it is unclear whether the levels of the mature form of NGF (the form with neurotrophic activity) were up-regulated in ASD children. Thus, another explanation for the observed NGF aberrations in ASD children is the compromise of proNGF to mature NGF conversion in the disease, which requires further investigations.

Abnormalities of neurotrophic factors were also thought to be associated with other neurological diseases, such as Alzheimer's disease and schizophrenia. Previous meta-analyses have demonstrated that blood BDNF levels were significantly decreased in patients with Alzheimer’s disease, whereas blood NGF and VEGF levels did not show significant differences between patients with Alzheimer’s disease and control subjects^82^. Additionally, meta-analyses showed that blood BDNF and NGF levels were significantly decreased in schizophrenia patients when compared with controls^83,84^. In contrast, blood VEGF levels were found not to be significantly different between first-episode schizophrenia patients and controls, whereas medicated multiple-episode schizophrenia had higher levels of VEGF than that of control subjects^85^. It is considered that ASD shares many molecular pathways with other neuropsychiatric diseases including schizophrenia. However, the present meta-analysis revealed heightened blood BDNF, NGF and VEGF levels in ASD children, it is very likely that patients with ASD have a unique neurotrophic factor profile comparing with other neuropsychiatric diseases, and this may partially explain the pathogenesis of ASD.

Although no significant between-study heterogeneity was found in the analysis of blood NGF and VEGF levels, one explanation for the low heterogeneity is that relatively few studies have been included. Additionally, we found high levels of heterogeneity among studies analyzing BDNF levels. Here we used subgroup and meta-regression analyses to adjust confounders that could explain the between-study heterogeneity. The potential moderators that we have analyzed including sampling source, assay type, sample size, publication year, age and gender did not address the heterogeneity. Obviously, the unexplained heterogeneity may due to other variables that we have not analyzed, such as medication status, disease severity and life style. However, the limited information on these variables in the included studies prevented us from analyzing whether the potential confounders had moderating effects on the outcome of the meta-analysis, therefore highlighting the need to control the variables in future studies.

Despite this work provides strong clinical evidence of the increased blood neurotrophic factor profile in children with ASD, there are still some limitations in this study. Firstly, the meta-analysis of peripheral blood neurotrophic factor levels in children with ASD and healthy controls produced a summary of results mainly from cross-sectional studies. Therefore, it is unclear whether the abnormal levels of neurotrophic factors are the cause or consequence for the development of ASD. Secondly, this meta-analysis analyzed blood neurotrophic factors levels in ASD children, but the neurotrophic factor profile in the brains of ASD children is largely unknown. The third limitation of the meta-analysis is that the effect size is very small, and the number of participants is very small except for BDNF and VEGF. In addition, a small number of studies evaluated NT-3 and NT-4, which may make it difficult to observe significant associations between the two neurotrophins and ASD. It should be noted that our meta-analysis showed a trend of decreased blood NT-3 levels in ASD children when compared with controls (*P* = 0.093). It is likely that we would observe a significant association between NT-3 and ASD with increased number of studies and sample size from future studies. Lastly, our study included only English articles, which may lead to publication bias. However, considering that we have excluded only one non-English articles, this is unlikely to have a significantly impact on the outcome of our meta-analysis.

In conclusion, the results of our meta-analysis showed the elevated peripheral blood BDNF, NGF and VEGF concentrations as a manifestation of children with ASD, strengthening the clinical evidence of an abnormal neurotrophic factor profile in children with ASD. Thus, future investigators into neurotrophic factors as potential therapeutic targets for the treatment of ASD are warranted.

## Method

This was an exploratory meta-analysis, and adhered to the guidelines that are recommended by the PRISMA statement (Preferred Reporting Items for Systematic reviews and Meta-Analysis)^86^.

### Search strategy and study selection

We have conducted a systematic search of peer-reviewed English articles using PubMed and Web of Science databases up to December 23, 2019. Our search strategy was:(neurotrophin OR neurotrophic factor OR brain-derived neurotrophic factor OR BDNF OR nerve growth factor OR NGF OR neurotrophin-3 OR NT-3 OR neurotrophin-4 OR NT-4 OR glial cell-derived neurotrophic factor OR GDNF OR insulin-like growth factor OR IGF OR vascular endothelial growth factor OR VEGF) AND (Autism), without year limitation. Additionally, we checked the reference list of relevant studies.

Original articles were screened according to the title and abstract, and then were scrutinized based on the following criteria: (1) measured peripheral blood neurotrophic factors; (2) neurotrophic factor that were available in three or more studies; (3) studies which provide the neurotrophic factor concentrations and standard deviation, or sample size and *P *value; (4) compared with matched healthy controls; (5) studies were excluded if the samples were from adult ASD patients, this is because neurotrophic factor levels were altered in adult^68^, which could have a confounding effect.

### Data extraction

Data from each included study was extracted by one investigator, and was verified by another investigator. Any inconsistencies were settled by discussions. Sample sizes, mean neurotrophic factor concentrations, standard deviation and *P *values were extracted as primary outcomes to generate effective size (ES). For studies that did not report neurotrophic factor concentrations, the sample size and *P* values were used to calculate the effect size^87^. Data on author last name, publication year, country of region, age, gender, sample source (serum or plasma sample or dried blood spot), diagnosis and assay type were also extracted.

### Statistical analysis

All statistical analyses were performed by comprehensive Meta-Analysis version 2 software. The ES was produced by sample size, mean concentration and standard deviation (SD), or by sample size and *P* value if the data of mean concentration were not available. The standardized mean difference of neurotrophic factor levels between children with ASD and healthy controls was calculated as ES, and converted into Hedge’s g statistic, which provides an unbiased adjusted ES for sample size^88^. We calculated ES estimates by evaluating each neurotrophic factor. And we used a random effects model in this meta-analysis, because if between-study heterogeneity is significant, the random effects model can produce a wider 95% confidence interval than the fixed effect model^89^.

We used the Cochrane Q test and I^2^ statistics to assess the heterogeneity among studies. *P* < 0.10 was considered statistically significant. The inconsistent levels among studies was decided by the I^2^ index to reflect the impact of heterogeneity, and an I^2^ index of 0.25, 0.50, 0.75 indicated low, moderate and high levels of heterogeneity, respectively^90^. We used subgroup analysis and unrestricted maximum-likelihood random-effects meta-regressions of ES to evaluate whether theoretically related covariates influence the outcome of meta-analysis. Publication bias was assessed via visual inspection of funnel plots, and Egger’s test was used to estimate the statistical significance.

We set all the statistical significances at *P* < 0.05 in this study except for where noted.

## Supplementary Information

Supplementary Information.
