# Supplementary material for: Peripheral blood neurotrophic factor levels in children with autism spectrum disorder: a meta-analysis
Source: Sci Rep. 2021 Jan 8;11:15. doi: 10.1038/s41598-020-79080-w (PMC7794512; doi:10.1038/s41598-020-79080-w)
Supplement: Supplementary file 1 — Supplementary Information. [file 41598_2020_79080_MOESM1_ESM.pdf]

# Peripheral Blood Neurotrophic Factor Levels in Children with Autism Spectrum Disorder: A Meta-analysis Study

Shu-Han Liu[1], Xiao-Jie Shi[1], Fang-Cheng Fan[1], Yong Cheng[1]*

[1]Center on Translational Neuroscience, College of Life and Environmental Sciences, Minzu University of China, 27 South Zhongguancun Avenue, Beijing 100081, China

* Corresponding to: Yong Cheng

**Table S1. Characteristics of Included Studies Measuring Neurotrophic Factor Concentrations**

| Study/Year | Measured | Country | Samples (ASD/HC) | Gender(% Male) (ASD/HC) | Mean Age (ASD/HC) | Sample Source | Diagnosis | Assay type | Mean (SD) Concentrations, ASD/HC* |
|---|---|---|---|---|---|---|---|---|---|
| Abdallah et al. 2013[1] | BDNF | Denmark | 359/741 | 81.06/80.3 | 0/0 | Dried blood spot | *ICD-8, ICD-10* | Luminex | P = 0.21 |
| Al-Ayadhi et al. 2011[2] | BDNF | Saudi Arabia | 46/53 | 89.1/86.8 | 7.4/8.4 | Serum | *DSM-IV* | ELISA | 353.2(78)/540.0(57) |
| Al-Ayadhi et al. 2012[3] | BDNF | Saudi Arabia | 44/40 | 93.2/95.0 | NA/NA | Serum | *DSM-IV*/CARS | ELISA | 0.38(0.067)/0.29(0.09) |
| Bryn et al. 2015[4] | BDNF | Norway | 65/30 | 78.5/46.7 | 11.7/11.3 | Plasma | *ICD-10* | ELISA | P=0.011 |
| Businaro et al. 2016[5] | BDNF | Italy | 18/29 | 94.4/93.1 | NA/NA | Serum | *DSM-IV-TR*/CARS | ELISA | 1.17(0.3727)/0.4604(0.14678) |
| Connolly et al. 2006[6] | BDNF | USA | 47/17 | 51.4/NA | 5.9/4.3 | Serum | *DSM-IV-TR* | ELISA | 32.28(24.29)/8.71(5.82) |
| Correia et al. 2010[7] | BDNF | Portugal | 146/50 | NA/NA | 7.1/7.5 | Platelet-rich plasma | ADI-R/ADOS-G | ELISA | 40.44(13.87)/23.26(12.34) |
| Croen et al. 2008[8] | BDNF | USA | 84/159 | 86.9/87.4 | 0/0 | Dried blood spot | *DSM-IV-TR* | Luminex | 0.0541(0.040)/0.0537(0.0477) |
| Enstrom et al. 2008[9] | BDNF | USA | 37/42 | 91/NA | 3.58/NA | Plasma | *DSM-IV*/*ICD-10*/ADI-R/ADOS | Luminex | 2.51(1.99)/1.46(1.13) |
| Francis et al. 2018[10] | BDNF | Athens | 45/26 | 86.7/57.7 | NA/NA | Serum | *DSM-IV*/ADI-R/ADOS-2/CARS | EIA system | 1.5949(0.7292)/3.48635(1.0091) |
| Gomez-Fernandez et al.2018[11] | BDNF | Italy | 54/54 | 83/84 | 3.6/4.1 | Plasma | *DSM- IV*/ADOS | Luminex | P=0.825 |
| Halepoto et al. 2015[12] | BDNF | Saudi Arabia | 60/25 | NA/NA | 6/7.04 | Serum | *DSM-IV*/CARS | ELISA | 0.39(0.24)/0.29(0.16) |
| Kasarpalkar et al. 2014[13] | BDNF | India | 48/25 | 52.1/NA | 7.4/7.44 | Serum | *DSM-IV* | ELISA | 250.56(92.70)/225.16(79.54) |
| Katoh-Semba et al.2007[14] | BDNF | Japan | 16/49 | NA/NA | NA/NA | Serum | NA | ELISA | 0.39(0.35)/0.35(0.13) |
| Mansour et al. 2010[15] | BDNF | Egypt | 20/20 | 75/75 | 6.93/6.87 | Serum | DSM-IV | ELISA | 1.25(0.77)/1.43(0.64) |
| Makkonen et al. 2011[16] | BDNF | USA | 13/15 | 92.3/86.7 | 8.7/8.7 | Serum | *ICD10*/CARS | ELISA | 6.330(6.591)/9.652(3.223) |
| Meng et al. 2017[17] | BDNF | China | 82/82 | 79.3/79.3 | 4.02/4.02 | Serum | *DSM*/CARS | ELISA | 17.75(5.43)/11.49(2.85) |
| Nelson et al. 2001[18] | BDNF | USA | 69/54 | 88.4/50.0 | 0/0 | Dried blood spot | *DSM III*/*DSM-IV* | RIC | 0.0374(0.0199)/0.0133(0.005) |

| | | | | | | | | | |
|---|---|---|---|---|---|---|---|---|---|
| Nelson et al. 2006[19] | BDNF | USA | 27/20 | NA/NA | 0/0 | Dried blood spot | *DSM-IV* | Luminex | 3.40(1.13)/3.30(0.84) |
| Ormstad et al. 2018[20] | BDNF | Norway | 65/30 | 80.0/46.7 | 11.2/10.9 | Serum | *ICD-10* | ELISA | 27.6(12.1)/21.2(9.6) |
| Ray et al. 2011[21] | BDNF | USA | 21/18 | 90.5/83.3 | 6.34/8.17 | Plasma | *DSM-IV*/ADI-R/CARS | ELISA | 15.86(6.92)/21.44(6.70)# |
| Ricci et al. 2013[22] | BDNF | Italy | 24/17 | 92/94 | 9.8/10.3 | Serum | *DSM-IV-TR*/CARS | ELISA | 0.88(0.34)/0.56(0.27) |
| Rodrigues et al. 2014[23] | BDNF | Brazil | 30/19 | 83.9/78.9 | 8/8 | Plasma | *DSM-IV-TR* | ELISA | P=0.28 |
| Skogstrand et al. 2019[24] | BDNF | Denmark | 801/2421 | 78.0/52.0 | 0/0 | Dried blood spot | *ICD-10* | ELISA | P=0.00135 |
| Taurines et al. 2014[25] | BDNF | Germany | 24/20 | 100/100 | 13.9/14.4 | Serum | *ICD-10*/SCQ/ADOS/ADI-R | ELISA | 20.61(6.04)/24.04(4.34) |
| Wang et al. 2015[26] | BDNF | China | 75/75 | 82.7/82.7 | 4/4 | Serum | *DSM-IV*/CARS | ELISA | 17.59(5.55)/11.21(2.79) |
| Zhang et al. 2014[27] | BDNF | China | 60/60 | 80/80 | 3.78/3.78 | Serum | *DSM-IV*/CARS | ELISA | P < 0.0001 |
| Dinçel et al. 2013[28] | NGF | Turkey | 49/49 | 79.6/55.1 | 8.38/7.34 | Serum | *DSM-IV* | ELISA | 0.04694(0.0514)/0.03294(0.01248) |
| Rodrigues et al. 2014[23] | NGF | Brazil | 30/19 | 83.9/78.9 | 8/8 | Plasma | *DSM-IV-TR* | ELISA | P=0.40 |
| Gomez-Fernandez et al.2018[11] | NGF | Italy | 21/16 | NA/NA | NA/NA | Serum | *DSM- IV*/ADOS | ELISA | P=0.05 |
| Nelson et al. 2001[18] | NT-3 | USA | 69/54 | 88.4/50.0 | 0/0 | Dried blood spot | *DSM III/DSM-IV* | Immunoassays | 0.0721(0.0408)/0.0764(0.04) |
| Nelson et al. 2006[19] | NT-3 | USA | 17/17 | NA/NA | 0/0 | Dried blood spot | *DSM-IV* | Luminex | 0.3023(0.074)/0.3639(0.0985) |
| Rodrigues et al. 2014[23] | NT-3 | Brazil | 30/19 | 83.9/78.9 | 8/8 | Plasma | *DSM-IV-TR* | ELISA | p=0.74 |
| Tostes MHFS et al.2012[29] | NT-3 | Brazil | 24/24 | 75/75 | 7.4/7.2 | Serum | *DSM-IV* | ELISA | 0.281(0.0423)/0.3953(0.0501) |
| Abdallah et al. 2013[1] | NT-4 | Denmark | 359/741 | 81.06/80.30 | 0/0 | Dried blood spot | *ICD-8, ICD-10* | Luminex | P=0.76 |
| Nelson et al. 2001[18] | NT-4 | USA | 69/54 | 88.4/50.0 | 0/0 | Dried blood spot | *DSM III/DSM-IV* | RIC | 0.0827(0.035)/0.0285(0.091) |
| Nelson et al. 2006[19] | NT-4 | USA | 27/24 | NA/NA | 0/0 | Dried blood spot | *DSM-IV* | Luminex | 0.1236(0.0773)/0.1316(0.0588) |
| Rodrigues et al. 2014[23] | NT-4 | Brazil | 30/19 | 83.9/78.9 | 8/8 | Plasma | *DSM-IV-TR* | ELISA | p=0.88 |

| | | | | | | | | | |
|---|---|---|---|---|---|---|---|---|---|
| Kajizuka et al. 2010[30] | VEGF | Japan | 31/31 | 100/100 | 12.3/12.4 | Serum | *DSM-IV-TR/ADI-R* | ELISA | 0.32(0.1979)/0.3138(0.2295) |
| Pecorelli et al. 2016[31] | VEGF | Italy | 12/8 | NA/NA | 17.7/15.4 | Serum | *DSM-IV/ADOS* | ELISA | 0.13959(0.09607)/0.16378(0.1174) |
| Skogstrand et al. 2019[24] | VEGF | Denmark | 801/2421 | 78/52 | 0/0 | Dried blood spot | *ICD-10* | ELISA | p=0.1321 |

*Mean concentrations were expressed as ng/ml except where noted; # Mean concentrations were expressed as ng/mg. only p values were available in some studies.

**Abbreviations:** ASD, Autism Spectrum Disorder; HC, Healthy Controls; BDNF, Brain-Derived Neurotrophic Factor; NGF, Nerve Growth Factor; NT-3, Neurotrophin-3; NT-4, Neurotrophin-4; VEGF, Vascular Endothelial Growth Factor; *ICD-8, International Classification of Diseases, Revision 8*; *ICD-10, International Classification of Diseases, Revision 10*; *DSM-IV, Diagnostic and Statistical Manual of Mental Disorders, fourth edition*; *DSM-IV-TR, Diagnostic and Statistical Manual of Mental Disorders, fourth edition, Text Revision*; *DSM-III, Diagnostic and Statistical Manual of Mental Disorders, third edition*; ADI-R, Autism Diagnostic Interview-Revised; ADOS, Autism Diagnostic Observation Schedule; ADOS-G, Autism Diagnostic Observation Schedule-Generic; CARS, Childhood Autism Rating Scale; SCQ, Social Communication Questionnaire; CSF, Cerebro-Spinal Fluid; ELISA, Enzyme-Linked ImmunoSorbent Assay; EIA, Emax Immuno Assay; RIC, Recycling Immunoaffinity Chromatography; NA, not available; SD, standard deviation.

# BDNF

| Group by Subgroup within study | Study name | Sample source | Statistics for each study | | | |
|---|---|---|---|---|---|---|
| | | | Hedges's g | Lower limit | Upper limit | p-Value |
| Dried blood spot | Abdallah et al. 2013 | Dried blood spot | -0.081 | -0.207 | 0.045 | 0.210 |
| Dried blood spot | Croen et al. 2008 | Dried blood spot | 0.000 | -0.264 | 0.264 | 1.000 |
| Dried blood spot | Nelson et al. 2001 | Dried blood spot | 1.567 | 1.163 | 1.972 | 0.000 |
| Dried blood spot | Nelson et al. 2006 | Dried blood spot | 0.097 | -0.472 | 0.665 | 0.739 |
| Dried blood spot | Skogstrand et al. 2019 | Dried blood spot | -0.131 | -0.211 | -0.051 | 0.001 |
| Dried blood spot | | | 0.279 | -0.376 | 0.934 | 0.404 |
| Plasma | Bryn et al. 2015 | Plasma | 0.568 | 0.131 | 1.005 | 0.011 |
| Plasma | Correia et al. 2010 | Plasma | 1.268 | 0.924 | 1.611 | 0.000 |
| Plasma | Enstrom et al. 2008 | Plasma | 0.653 | 0.204 | 1.103 | 0.004 |
| Plasma | Gomez-Fernandez et al. 2018 | Plasma | -0.042 | -0.417 | 0.332 | 0.825 |
| Plasma | Ray et al. 2011 | Plasma | -0.802 | -1.443 | -0.160 | 0.014 |
| Plasma | Rodrigues et al. 2014 | Plasma | 0.315 | -0.254 | 0.884 | 0.277 |
| Plasma | | | 0.348 | -0.266 | 0.962 | 0.267 |
| Serum | Al-Ayadhi et al. 2011 | Serum | -2.744 | -3.291 | -2.196 | 0.000 |
| Serum | Al-Ayadhi et al. 2012 | Serum | 1.132 | 0.674 | 1.589 | 0.000 |
| Serum | Businaro et al. 2016 | Serum | 2.717 | 1.920 | 3.515 | 0.000 |
| Serum | Connolly et al. 2006 | Serum | 1.102 | 0.522 | 1.682 | 0.000 |
| Serum | Francis et al. 2018 | Serum | -2.222 | -2.823 | -1.620 | 0.000 |
| Serum | Halepoto et al. 2015 | Serum | 0.451 | -0.017 | 0.918 | 0.059 |
| Serum | Kasarpalkar et al. 2014 | Serum | 0.284 | -0.196 | 0.765 | 0.247 |
| Serum | Katoh-Semba et al. 2007 | Serum | 0.193 | -0.366 | 0.751 | 0.499 |
| Serum | Mansour et al. 2010 | Serum | -0.249 | -0.859 | 0.361 | 0.423 |
| Serum | Makkonen et al. 2011 | Serum | -0.637 | -1.377 | 0.103 | 0.092 |
| Serum | Meng et al. 2017 | Serum | 1.437 | 1.095 | 1.779 | 0.000 |
| Serum | Ormstad et al. 2018 | Serum | 0.558 | 0.121 | 0.994 | 0.012 |
| Serum | Ricci et al. 2013 | Serum | 1.002 | 0.355 | 1.649 | 0.002 |
| Serum | Taurines et al. 2014 | Serum | -0.631 | -1.228 | -0.033 | 0.038 |
| Serum | Wang et al. 2015 | Serum | 1.445 | 1.087 | 1.803 | 0.000 |
| Serum | Zhang et al. 2014 | Serum | 0.731 | 0.363 | 1.098 | 0.000 |
| Serum | | | 0.293 | -0.089 | 0.675 | 0.133 |
| Overall | | | 0.302 | 0.012 | 0.593 | 0.041 |

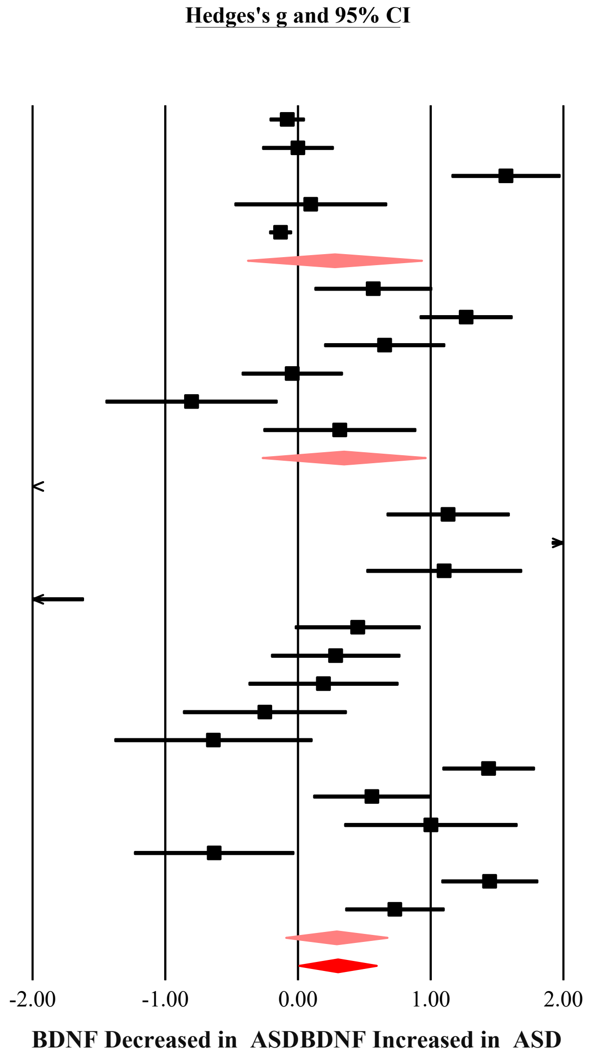


**Figure S1. Sub-group Analysis Stratified by Samples Source for BDNF**

Forrest plot showing pooled results comparing brain-derived neurotrophic factor levels between autism spectrum disorder (ASD) and healthy controls stratified by source of sampling (dried blood spot, plasma and serum). The sizes of the squares are proportional to study weights. Diamond marker indicates pooled effect size. CI, confidence interval.

# BDNF

| Group by<br>Subgroup within study | Study name | Assay type | Statistics for each study | | | |
|---|---|---|---|---|---|---|
| | | | Hedges's g | Lower limit | Upper limit | p-Value |
| ELISA | Al-Ayadhi et al. 2011 | ELISA | -2.744 | -3.291 | -2.196 | 0.000 |
| ELISA | Al-Ayadhi et al. 2012 | ELISA | 1.132 | 0.674 | 1.589 | 0.000 |
| ELISA | Bryn et al. 2015 | ELISA | 0.568 | 0.131 | 1.005 | 0.011 |
| ELISA | Businaro et al. 2016 | ELISA | 2.717 | 1.920 | 3.515 | 0.000 |
| ELISA | Connolly et al. 2006 | ELISA | 1.102 | 0.522 | 1.682 | 0.000 |
| ELISA | Correia et al. 2010 | ELISA | 1.268 | 0.924 | 1.611 | 0.000 |
| ELISA | Halepoto et al. 2015 | ELISA | 0.451 | -0.017 | 0.918 | 0.059 |
| ELISA | Kasarpalkar et al. 2014 | ELISA | 0.284 | -0.196 | 0.765 | 0.247 |
| ELISA | Katoh-Semba et al. 2007 | ELISA | 0.193 | -0.366 | 0.751 | 0.499 |
| ELISA | Mansour et al. 2010 | ELISA | -0.249 | -0.859 | 0.361 | 0.423 |
| ELISA | Makkonen et al. 2011 | ELISA | -0.637 | -1.377 | 0.103 | 0.092 |
| ELISA | Meng et al. 2017 | ELISA | 1.437 | 1.095 | 1.779 | 0.000 |
| ELISA | Ormstad et al. 2018 | ELISA | 0.558 | 0.121 | 0.994 | 0.012 |
| ELISA | Ray et al. 2011 | ELISA | -0.802 | -1.443 | -0.160 | 0.014 |
| ELISA | Ricci et al. 2013 | ELISA | 1.002 | 0.355 | 1.649 | 0.002 |
| ELISA | Rodrigues et al. 2014 | ELISA | 0.315 | -0.254 | 0.884 | 0.277 |
| ELISA | Skogstrand et al. 2019 | ELISA | -0.131 | -0.211 | -0.051 | 0.001 |
| ELISA | Taurines et al. 2014 | ELISA | -0.631 | -1.228 | -0.033 | 0.038 |
| ELISA | Wang et al. 2015 | ELISA | 1.445 | 1.087 | 1.803 | 0.000 |
| ELISA | Zhang et al. 2014 | ELISA | 0.731 | 0.363 | 1.098 | 0.000 |
| ELISA | | | 0.404 | 0.024 | 0.785 | 0.037 |
| no ELISA | Abdallah et al. 2013 | no ELISA | -0.081 | -0.207 | 0.045 | 0.210 |
| no ELISA | Croen et al. 2008 | no ELISA | 0.000 | -0.264 | 0.264 | 1.000 |
| no ELISA | Enstrom et al. 2008 | no ELISA | 0.653 | 0.204 | 1.103 | 0.004 |
| no ELISA | Francis et al. 2018 | no ELISA | -2.222 | -2.823 | -1.620 | 0.000 |
| no ELISA | Gomez-Fernandez et al. 2018 | no ELISA | -0.042 | -0.417 | 0.332 | 0.825 |
| no ELISA | Nelson et al. 2001 | no ELISA | 1.567 | 1.163 | 1.972 | 0.000 |
| no ELISA | Nelson et al. 2006 | no ELISA | 0.097 | -0.472 | 0.665 | 0.739 |
| no ELISA | | | 0.015 | -0.618 | 0.647 | 0.964 |
| Overall | | | 0.301 | -0.025 | 0.627 | 0.070 |

**Hedges's g and 95% CI**

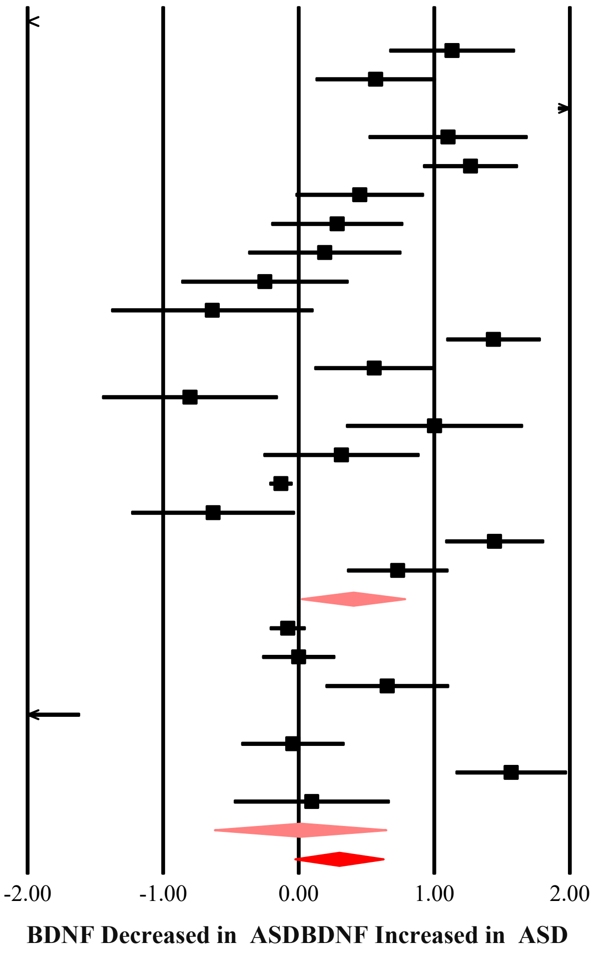


**Figure S2. Sub-group Analysis Stratified by Assay Type for BDNF**

Forrest plot showing pooled results comparing brain-derived neurotrophic factor levels between autism spectrum disorder (ASD) and healthy controls stratified by assay type (ELISA and no ELISA). The sizes of the squares are proportional to study weights. Diamond marker indicates pooled effect size. CI, confidence interval

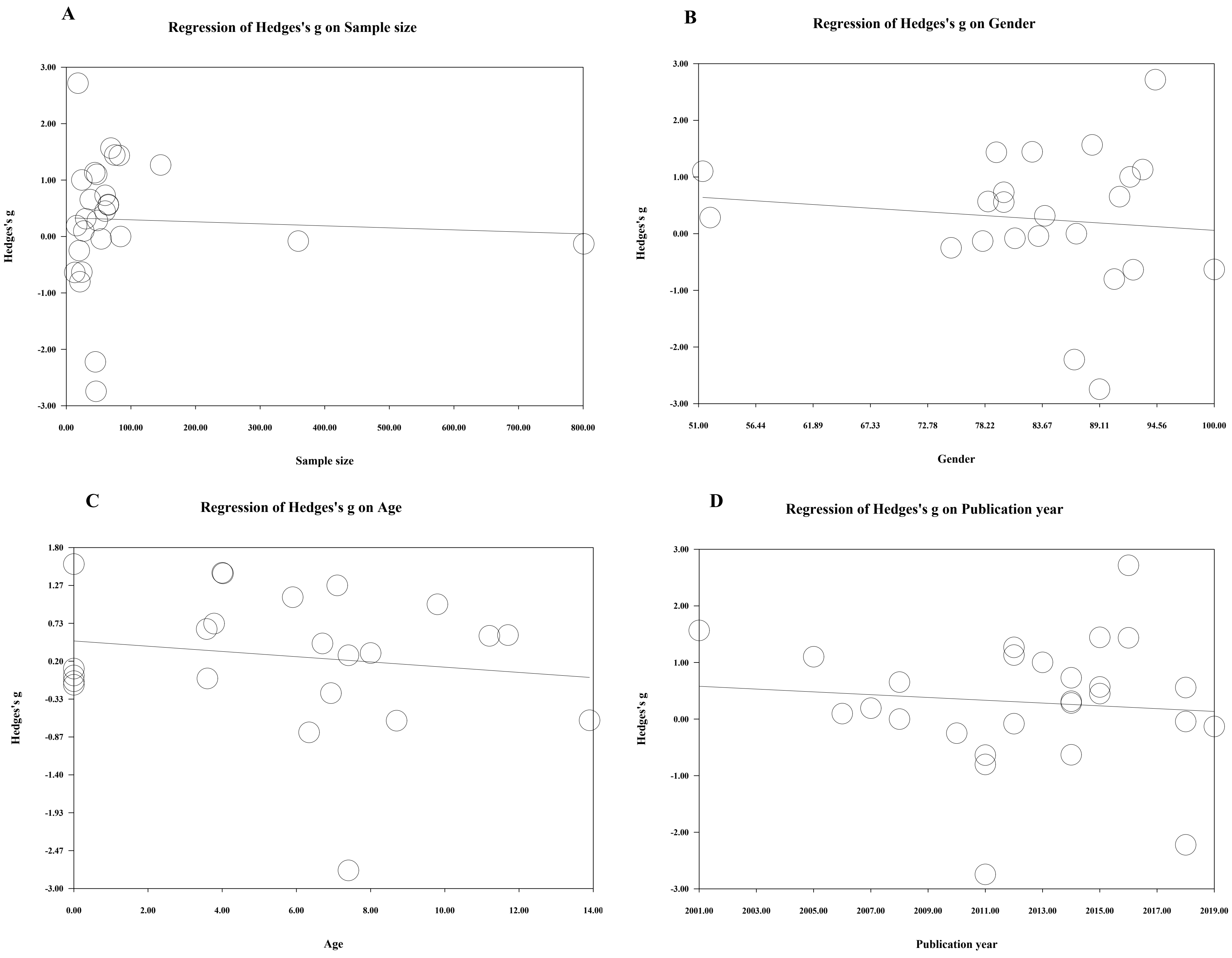


**Figure S3. Meta-Regression**

Association between sample size (A), gender of patients (B) ,age of patients (C) or publication year (D) and effect size (Hedges's g) for BDNF. The sizes of the circles are proportional to study weights.

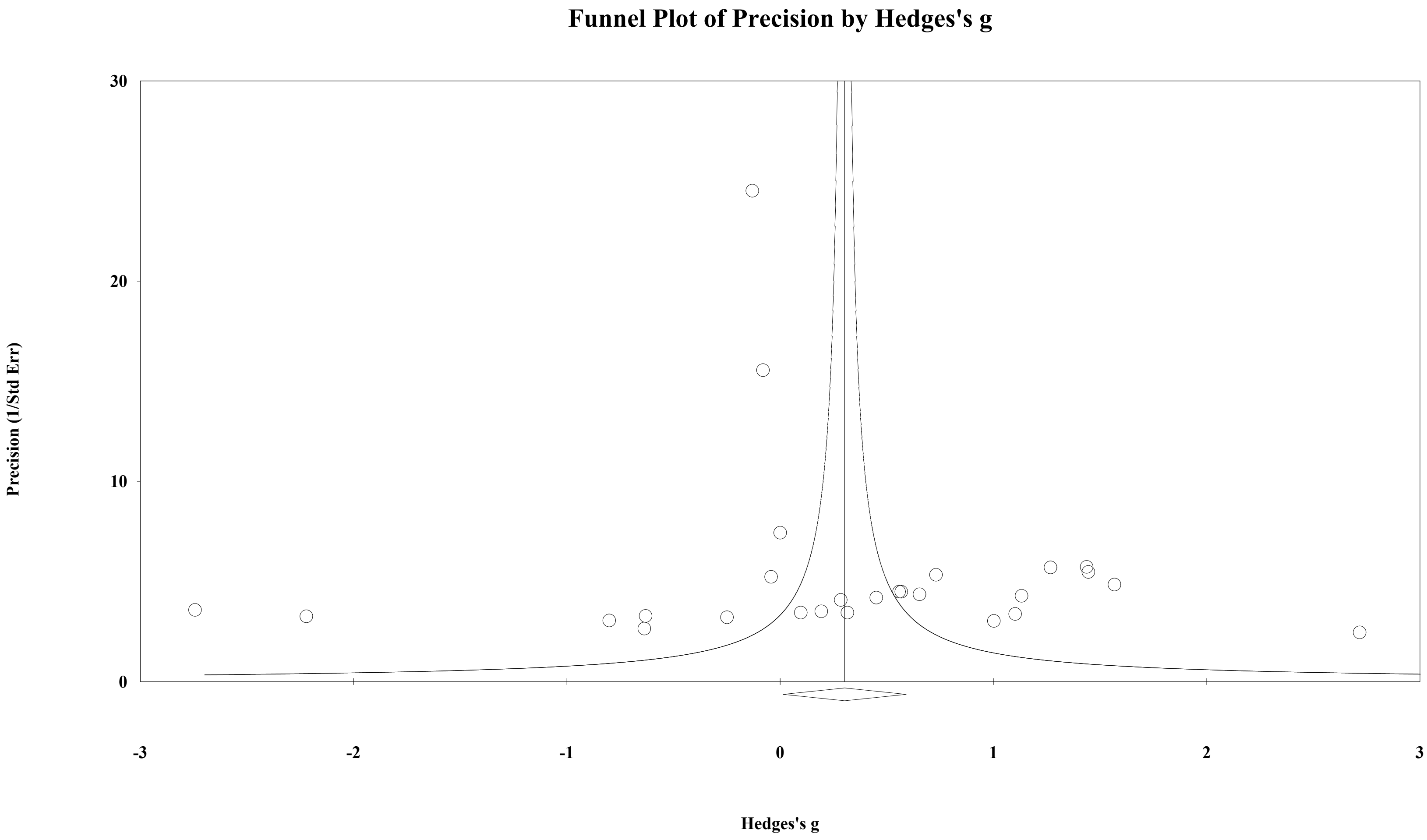


**Figure S4. Funnel Plot of Precision by Hedges g Statistics**

Publication bias in studies comparing brain-derived neurotrophic factor levels between children with autism spectrum disorder (ASD) and healthy controls. The plots describe the effect size (Hedges g statisic) of studies against their precision (inverse of SE). Data markers indicate individual studies. Diamond marker indicates pooled effect.
